# Effect of Freezing Temperature on the Thermal, Rheological, and Gelatinization Properties of Freeze-Thaw-Dehydrated Potato Powder

**DOI:** 10.3390/gels10110744

**Published:** 2024-11-15

**Authors:** Xinyan Duan, Tingting Zhang, Qiannan Liu, Liang Zhang, Wei Liu, Ruixuan Zhao, Honghai Hu

**Affiliations:** Institute of Food Science and Technology, Chinese Academy of Agricultural Sciences, Comprehensive Key Laboratory of Agro-Products Processing, Ministry of Agriculture and Rural Affairs, Beijing 100193, China

**Keywords:** freeze-thaw, potato powder gel, thermal property, gel property, correlation analysis

## Abstract

To promote the application of freeze-thaw-dehydrated (FTD) potatoes and their gels, this study aimed to investigate the effects of freezing temperature on the physicochemical and gel properties of FTD potato powder and their correlation. The results revealed that, as the freezing temperature decreased, the solubility exhibited an overall downwards trend resulting from soluble solids and amylose liberation. Owing to the better cell integrity at −20 °C, the solubility was greater than that of the other treatment groups. In contrast, the trough viscosity and melting enthalpy increased, and the final viscosity, and setback first increased but then decreased. Regarding the properties of the FTD potato powder gel, the storage modulus, loss modulus, hardness, adhesiveness, chewiness, and consistency first increased but then decreased with decreasing freezing temperature. At a moderate freezing temperature (−20 °C), the solubility and stability of the FTD potato powder were well maintained, and the final viscosity, setback, and hardness reached their highest values. Correlation analysis revealed that, with decreasing freezing temperature, the amount of FTD potato powder initially increased, followed by a decrease in the final viscosity and setback. This trend was positively correlated with the hardness of the FTD potato gel (r = 0.98, r = 0.93).

## 1. Introduction

Potato is the fourth most important food crop worldwide and a staple food in many countries [1,2]. In addition, potatoes can also be used to produce noodles [3], vermicelli [4], potato chips [5], and other products. Chuño, a traditional freeze-thaw-dehydrated (FTD) processed potato, originated in the ancient Andes (400 B.C.). The chuño process involves freezing harvested potatoes at low night temperatures and allowing them to thaw naturally during the day for approximately four days. The potatoes are subsequently stepped on for the skin to peel off and then left on the ground for 2–3 weeks [2,6]. The chuño process allows food production for long-term storage and emergencies [7]. In addition, it greatly reduces the cost of long-distance transportation. Therefore, it is very popular in the Andean region and has great market potential.

The process of preparing traditional FTD potatoes requires a sufficiently low temperature at night to freeze and a sufficiently warm temperature during the day to thaw. Even minor fluctuations in freezing temperature can substantially influence the physical, chemical, and gel properties of the resulting product [8,9]. However, temperature fluctuations in natural environments are often unpredictable and beyond human control. As a result, the quality of FTD potatoes produced in a natural environment fluctuates greatly, and the processing characteristics of their gel products are also unstable. This greatly limits the application and development of FTD potatoes. Therefore, it is necessary to explore the effects of freezing temperature on the physicochemical and gel properties of FTD potato powder to promote its application.

Controlling the size and number of ice crystals is the key to freezing. The formation of ice crystals largely depends on the freezing rate and temperature [10,11,12]. In potatoes, the different sizes of ice crystals formed during the freezing process alter the microstructure, physicochemical properties, and crystallization state of starch [13,14]. In addition, as the water formed by ice crystals melts, dehydration contributes to the broken cellular structure [15]. After FTD potato powder processing, the potato protein and zinc contents decrease, the calcium content increases, and the iron content remains unchanged [7]; the content of antioxidants decreases but is far from being eliminated [6]; and the apparent amylose content and ΔH values decrease, but the apparent viscosity increases [16]. In conclusion, research has focused mainly on the changes in components and physicochemical properties after FTD treatment. However, during FTD potato powder processing, the effects of freezing temperature on the physicochemical and processing properties of potatoes, and the relationship between physicochemical properties and processing characteristics, are rarely reported. According to our previous research, freezing treatment affects the cellular microstructure and reduces the protein and mineral contents due to damage to the potato cell wall and membrane. However, at a moderate freezing rate (freeze at −20 °C), the cells remain relatively intact, with high retention of protein and minerals [17]. As a continuation and expansion of previous research, further exploration of the impact of freezing temperature on the physicochemical and processing properties of FTD potatoes is crucial.

Therefore, this study aimed to investigate the effects of freezing temperature on the physicochemical properties (solubility, swelling power, thermodynamic properties, and pasting properties) and gel characteristics (rheological properties and gel texture) of FTD potato powder. Moreover, the relationships between the physicochemical properties and gel characteristics of the FTD potato powder were elucidated via correlation coefficient analysis. The findings of this study provide a theoretical basis for the further application of FTD in potato and its gel products.

## 2. Results and Discussion

### 2.1. Solubility and Swelling Power of the FTD Potato Powder

**Figure 1:**
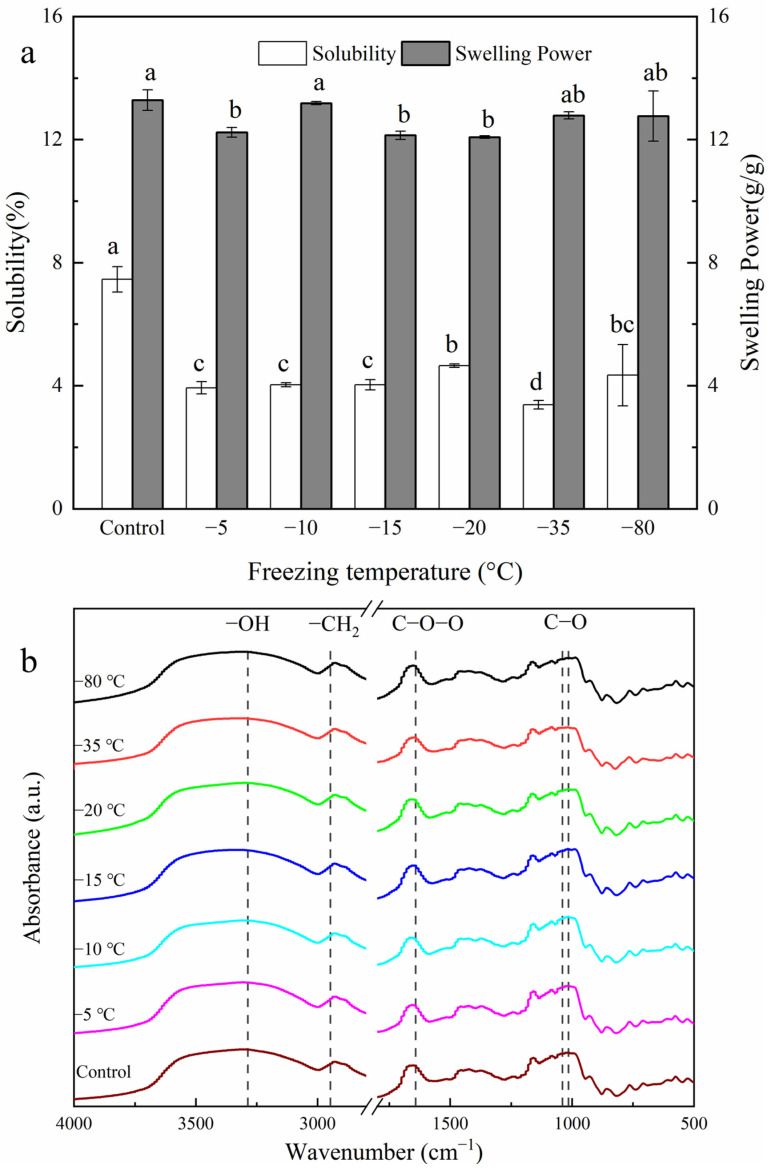
Solubility, swelling power (**a**), and FTIR spectra (**b**) of FTD potato powder at different freezing temperatures. The bars denoted with different lowercase letters indicated significant differences (*p* < 0.05).

The swelling power of potato powder on the basis of its solubility under different freezing treatments is shown in Figure 1a. The solubility of the potato powder in the control group was significantly greater than that in the various freeze-thaw treatments. The highest solubility (4.65) of the freeze-treated samples was recorded at −20 °C. In addition, the solubility was correlated with the soluble solids in the samples under the different freeze treatments. According to our previous research, FTD treatment causes protein and mineral losses, and the protein retention rate ranges from 65.16 to 83.66%, reaching a peak at −20 °C [17]. The loss of soluble solids (protein and mineral) reduced the solubility, hence, compared with the control group, the highest content of soluble solids at −20 °C indicated the highest solubility. Studies have shown that the use of magnetic fields, ultrasonication and microwaves can reduce structural damage, resulting in less protein and mineral outflows [18,19].

During swelling, the double helices in amylose melt [20], and the molecules are separated and recrystallized. As a result, the amylopectin phosphate groups are mutually exclusive, increasing the contact area between starch and water molecules. This induces further starch hydration, resulting in the formation of a polymer [21]. In the present study, the swelling powers of the −5, −15, and −20 °C FTD treatment groups were significantly lower than those of the control group. The reduced swelling power might be due to molecular rearrangement in the starch granules after FTD treatment. The molecular rearrangement increased the interaction between starch molecules, weakening their ability to combine with water molecules and leading to weakening of the swelling power [22,23,24]. In addition, the interactions between amylose and amylopectin reduce the number of free hydroxyl groups that interact with water molecules. The swelling power did not decrease, which can be attributed to damage to starch particles, which formed grooves and channels on starch molecules, increasing their water-binding capacity.

### 2.2. Thermal Characteristics of the FTD Potato Powder

The thermal characteristics of potato powder following different freeze treatments are presented in Table 1. Compared with that of the control group, the onset melting temperature (T_o_) of the potato powder after FTD treatment differed significantly, except at −80 °C. The control group presented the highest T_o_, whereas the −35 °C group presented the lowest T_o_ Moreover, compared with that of the control group, the conclusion melting temperature (T_c_) at −15 °C significantly increased, and the melting enthalpy (ΔH) of the respective freeze treatments significantly increased. Freeze-thaw treatments destroyed the integrity of the cells, making it easier for water molecules to penetrate starch molecules. This hinders recrystallization, which is characterized by a reduced initial gelatinization temperature [25]. In addition, freeze-thaw treatments had different effects on starch granules, given the variation in the location and size of the ice crystals. ΔH represents the energy required for melting of the amylopectin crystallites (change from a crystalline to a noncrystalline structure), destroying the double helix structure [26]. In this study, the ΔH values of the potato powder significantly increased after FTD treatment, implying that more energy was required during gelatinization. This finding indicates that it was more difficult for the potato powder to gelatinize after freeze-thaw treatment, possibly because of the increased amylopectin content caused by amylose dissolution.

### 2.3. ATR-FTIR of the FTD Potato Powder

The short-range ordered structure of starch was revealed at an absorbance ratio of 1047/1022 cm^−1^ (R 1047/1022), which was related to alterations in the double helix structure [27]. Similarly, an asymmetrical stretching peak at 1635 cm^−1^ and a weak symmetrical stretching peak near 1401 cm^−1^ were assigned to −COO absorbance [28]. In addition, the absorption peak at 2928 cm^−1^ is related to the antisymmetric stretching vibrations of −CH_2_, which could increase the lipophilicity of the raw materials. The extremely wide absorption peak in the range of 3200–3500 cm^−1^ is also attributed to the stretching vibrations of the hydroxyl groups present in the water and sugar rings in the samples [29].

The ATR-FTIR spectra of the FTD and control potato powder samples are shown in Figure 1b. The position of the characteristic absorption peak was not significantly different from that of the control, implying that freeze-thaw dehydration at different temperatures did not alter the chemical groups or lead to the formation of new chemical groups. The absorbance ranged from 3200 to 3500 cm^−1^ and decreased slightly with decreasing freezing temperature. Similarly, the short-range ordering was not significantly altered. This is possibly because potato cell damage is accompanied by the loss of the double helices in the amylopectins, hence decreasing starch short-range ordering [30]. Alternatively, FTD treatment could cause amylose dissolution, rearranging the double helix structure of amylopectin while increasing the short-range order [13].

### 2.4. Pasting Properties of the FTD Potato Powder

The pasting properties of the potato powder under the different treatments are shown in Table 2. When the starch is heated and sheared, the granules are destroyed. As a result, the amylose is leached first, followed by amylopectin [31]. In addition, as the temperature decreases, the viscosity reaches the final viscosity because of the reassociation and short-term retrogradation of the amylose molecules [32].

In the present study, the peak, trough, final viscosity, and setback values significantly increased compared with those of the control. Moreover, the breakdown significantly decreased, whereas the pasting temperature did not significantly differ from that of the control (Table 2). The peak viscosity and breakdown value were lowest at −20 °C. The subsequent increase in viscosity could result from the protein and dietary fibres inhibiting the swelling power of starch lost during the FTD treatments [33]. Second, freeze-thaw treatments destroyed the internal structure of the starch granules, increased the number of voids on the starch granules, and increased the contact area between the water and starch, which made it easier for the water molecules to enter the starch granules, resulting in increased viscosity [34]. Thus, the potato powder treated at −20 °C had relatively high cell integrity and protein content, resulting in a relatively low viscosity.

Herein, the setback value is the difference between the final viscosity and trough viscosity. The breakdown value is the difference between the peak viscosity and the trough viscosity, which reflects the anti-aging ability of starch [35]. The loss of protein following the freezing treatments increased access to starch molecules, which implies that the FTD treatments were prone to aging. In addition, the pasting temperature was generally negatively correlated with the relative crystallinity. The absorbance ratio of 1047/1022 cm^−1^ (R 1047/1022) in the FTIR spectrum did not significantly change; moreover, there was no significant change in the pasting temperature. This finding also indicated that the degree of structural ordering was not affected by the FTD treatments.

### 2.5. Rheological Properties of the FTD Potato Powder Gel

#### 2.5.1. Strain Sweep Analysis

**Figure 2:**
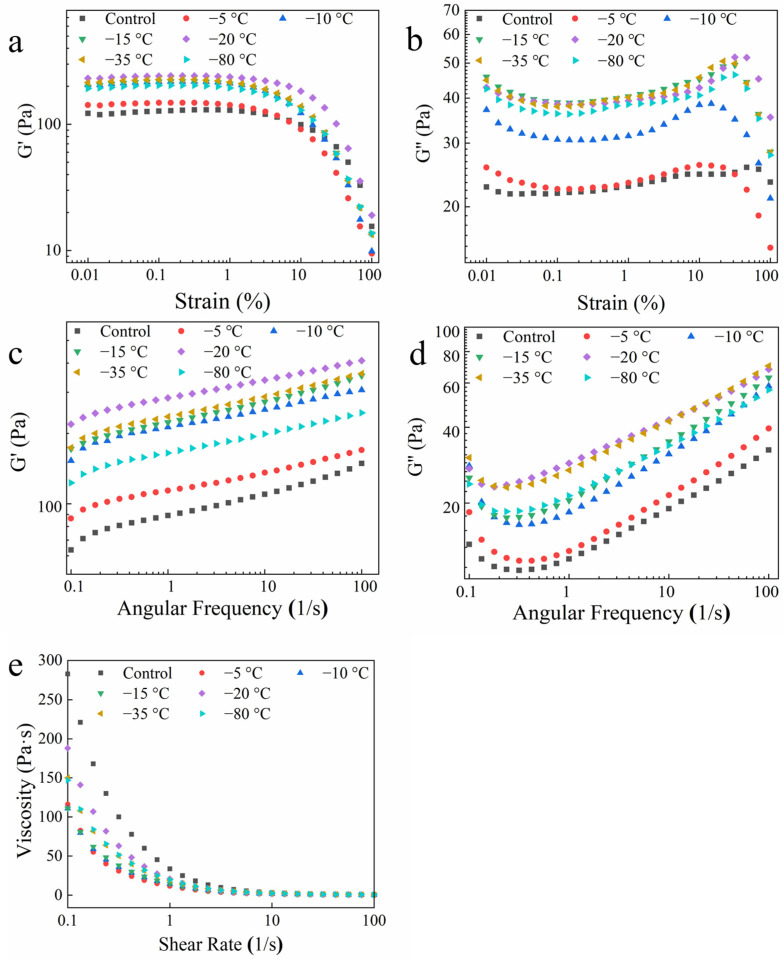
Rheological properties of FTD potato powder at different freezing temperatures: the strain sweep (**a**,**b**), frequency sweep (**c**,**d**), and steady shear (**e**).

The strain sweeps of the potato powder under the different freeze treatments are depicted in Figure 2a,b. With increasing strain, the storage modulus (G′) and loss modulus (G″) remained almost constant in the linear viscoelastic range (LVR). However, after exceeding the LVR, G′ suddenly decreased, whereas G″ first steadily increased but then suddenly decreased. The LVR of the potato powder after FTD treatment at different freezing temperatures is presented in Table 1. Notably, the LVR values following the FTD treatments were lower than those of the control. Among them, the LVR value of potato powder frozen at −5 °C was the lowest, whereas that at −20 °C was the highest, with a noticeable increasing trend at first and then a decline. The structure of the natural potato powder was relatively stable, and the FTD treatments destroyed the internal structure of the potato powder. Both higher and lower freezing temperatures significantly damage the cell. However, at −20 °C, the cell integrity was the highest [17], implying that the LVR value was the highest at −20 °C. 

#### 2.5.2. Frequency Sweep Analysis

G′ reflects the sample elasticity after deformation, and G″ is the energy lost while resisting viscous resistance. G′ > G″ at the same frequency across all the treatments revealed that the solid properties of the potato powder were greater than the liquid properties, with obvious elastic properties (Figure 2c,d). This implies a strong network structure [36]. Notably, the G′ and G″ values across all the treatments increased with increasing frequency. Moreover, the G′ and G″ values after the FTD treatments were greater than those of the control group. The maximum G′ and G″ values were recorded at −20 °C, suggesting that the viscoelasticity of potato powder was optimal at −20 °C.

#### 2.5.3. Steady Rheology Analysis

The apparent viscosity of potato powder rapidly decreased with increasing shear rate, followed by gradual stabilization (Figure 2e), implying that shear thinning took place. The shear thinning can be attributed to weakened intermolecular forces caused by shear, such as breaking the hydrogen bond and double helix [37]. Additionally, the R2 values of the fitting equations (Table 1) were greater than 0.99, indicating that the fitting equations were significantly correlated with the rheological curve. The flow behavior index across all the treatments was less than 1, implying that the potato powder was a pseudoplastic fluid and was characterized by shear thinning [38]. Moreover, the consistency coefficient (K) value of the FTD samples was lower than that of the control. Notably, the K value first decreased, then increased, and finally decreased again with decreasing freezing temperature. Among the different freezing treatments, −5 °C had the most significant effect on the flow behavior index. Generally, the FTD treatment decreased the K value, indicating a decrease in viscosity. Thus, the K values were related to the degree of damage to the FTD potato powder cells. For the treatment group, the cell integrity was highest at −20 °C and, correspondingly, the apparent viscosity was also highest.

### 2.6. Textural Properties of the FTD Potato Powder Gel

The potato powder texture characteristics following FTD treatment are shown in Table 3. The textural characteristics of gels reflect their shape and food taste. The hardness, adhesiveness, and chewiness of the gel in the control group were significantly lower than those in the FTD treatment groups. The hardness, resilience, elasticity, adhesiveness, and chewiness among the treatment groups first increased but then decreased with decreasing freezing temperature. The gel hardness reached a maximum at −15 and −20 °C. This is consistent with findings from previous studies, where the hardness value was the peak pressure recorded by a texture analyser probe during the first stamping, and it was affected by amylose gelation [39]. In addition, adhesiveness is the separation force required for the probe to break away from the contact surface. Chewiness is the energy required for chewing a semisolid material to a swallowable state. The increased hardness and chewiness are attributed to a higher final viscosity and setback caused by the further combination of starch molecules due to the destruction of the starch granules. At moderate freezing temperatures, the cell wall and membrane are well preserved. In contrast, cell integrity decreases, and proteins and other components are lost at relatively high and low freezing temperatures, resulting in decreased hardness. Research has revealed that the highest integrity is maintained at −20 °C [17]. Elasticity and resilience exhibited similar trends. Freezing treatment induces amylose dissolution, increasing the proportion of amylopectin, which is important for adhesiveness.

### 2.7. Correlation Analysis

**Figure 3:**
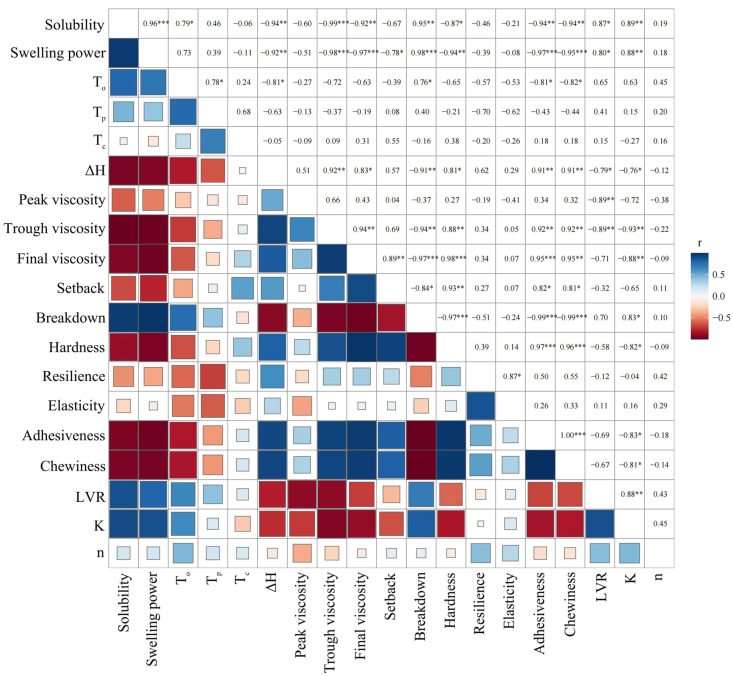
Correlation analysis of FTD potato powder at different freezing temperatures. *** Significant at *p* < 0.001, ** Significant at *p* < 0.05, * Significant at *p* < 0.01. Blue represents positive correlation and red represents negative correlation. The darker the color, the larger the square, and the stronger the correlation.

The correlation between the physicochemical properties and processing characteristics of the FTD potato powder is shown in Figure 3. Hardness was positively correlated with trough viscosity (r = 0.88), final viscosity (r = 0.98), setback (r = 0.93), and ΔH (r = 0.81), and negatively correlated with breakdown (r= −0.97), swelling power (r = −0.94), and solubility (r = −0.87). Adhesiveness was positively correlated with trough viscosity (r = 0.92), final viscosity (r = 0.95), setback (r = 0.82), and ΔH (r = 0.91). Chewiness was positively correlated with trough viscosity (r = 0.92), final viscosity (r = 0.95), setback (r = 0.81), and ΔH (r = 0.91). Adhesiveness was also positively correlated with trough viscosity (r = 0.92), final viscosity (r = 0.95), setback (r = 0.82), and ΔH (r = 0.91). LVR was positively correlated with solubility (r = 0.87) and swelling power (r = 0.80), and negatively correlated with peak viscosity (r = −0.89) and trough viscosity (r = −0.89). K was also positively correlated with solubility (r = 0.89), swelling power (r = 0.88), and breakdown (r = 0.83), and negatively correlated with trough viscosity (r = −0.93), final viscosity (r = −0.88), and ΔH (r = −0.76). Viscosity and hardness are related to reduced cell integrity and protein and fibre contents [33,40]. Compared with those of the control group, the cell integrity and protein content decreased after freezing, leading to increased molecular interactions. A further setback and final viscosity caused an increase in the hardness. Additionally, cell integrity destruction and amylose dissolution increase the amylopectin proportion, which increases adhesiveness, chewiness, and ΔH [41]. LVR and K were positively correlated with solubility and swelling power, possibly because cell damage and protein loss were related to decreased solubility and swelling power, which negatively affected the LVR and K values. Generally, cell damage results in increased viscosity, which reduces the LVR and K values.

## 3. Conclusions

Freezing temperatures impact the physicochemical, rheological, and gelatinization properties of FTD potato powder. FTD treatment induces the loss of soluble solids in potatoes, reducing their solubility. As the freezing temperature decreased, the ΔH and trough viscosity of the FTD potato powder increased, whereas the final viscosity and setback initially increased but subsequently decreased. The maximum solubility and viscosity of the FTD treatment is at −20 °C. FTD treatment does not alter or form new chemical groups, nor does it affect structural ordering. However, the dynamic modulus, hardness, adhesiveness, and chewiness of the potato powder significantly increased. In addition, the viscoelasticity, hardness, adhesiveness, and chewiness peaked at −20 °C. Correlation analysis revealed that hardness is positively correlated with trough viscosity, final viscosity, setback, and ΔH, but negatively correlated with solubility, swelling power, and breakdown. Adhesiveness and chewiness are also positively correlated with trough viscosity, final viscosity, setback, and ΔH. The findings from this study provide a theoretical basis for the further application of FTD potato powder and gels. FTD treatment inevitably causes the loss of nutrients, so determining what treatment can reduce the loss of nutrients is also a direction of future research. In addition, the further application of potato powder and potato gel treated with FTD needs to be studied.

## 4. Materials and Methods

### 4.1. Materials

The potatoes (Atlantic) were purchased from Rongteng Science and Technology Development Co., Ltd. (Jinchang, China) and stored at 4 °C in a refrigerator (Sanyo Electric Co., Ltd, Osaka, Japan) until subsequent analysis. All chemicals and reagents were of at least analytical grade.

### 4.2. FTD Treatment

The potato tubers were peeled and cut into 1.5 cm × 1.5 cm × 1.5 cm cubes. Next, the 50 potato cubes were cooled at 4 °C for 4 h and frozen at −5, −10, −15, −20, −35, and −80 °C for 12 h in a refrigerator. For the control group, the potato cubes were maintained at 4 °C. Next, the frozen cubes were thawed at 4 °C for 4 h and then centrifugally dehydrated for 5 min [17]. The dehydrated potato cubes were dried at 50 °C for 6 h in a drying oven (DGG-9203A drying oven, Shanghai, China) and ground with an IKA A11 basic grinder, after which the powder was passed through a 60-mesh sieve and packaged in zip lock bags for further research.

### 4.3. Solubility and Swelling Power Analyses

A total of 20 mL of water and 1.0 g of potato powder (dry weight) were added to a 50 mL centrifuge tube, heated at 95 °C for 30 min, and then cooled to room temperature. The samples were then centrifuged at 8000× *g* for 30 min. The supernatant was transferred to an aluminum box and dried to a constant weight at 105 °C. Finally, the solubility and swelling power were calculated via the following equations [42]:

$$WS\;\left(\%\right)=\frac{m_{u}}{m}\times 100\%$$

$$SP\left(\%\right)=\frac{m_{1}-m_{2}}{m\times \left(100\%-WS\%\right)}$$

where *WS* (%) represents the solubility; *m_u_* and *m* (g) represent the weights of dry matter in the supernatant and sample, respectively; *SP* (%) represents the swelling power; and *m*_1_ and *m*_2_ (g) represent the weights of the tube and sample before and after centrifugation, respectively.

### 4.4. Thermal Characteristics Measurement

The thermal characteristics of the potato powder were analysed via a DSC 8000 differential scanning calorimeter (PerkinElmer Instrument Co., Ltd., Shanghai, China) according to a previously described method [43]. The samples were heated from 25 to 110 °C at a rate of 5 °C/min. For each sample, T_o_, T_p_, T_c_, and ΔH were obtained.

### 4.5. Fourier Transform Infrared Spectroscopy (FTIR)

The FTIR spectra were developed to assess the functional group and short-range ordering changes via a Thermo Nicolet 67 FTIR instrument (Thermo Fisher Scientific Inc., Shanghai, China). Briefly, 2 mg of potato sample was ground with KBr at a ratio of 1:100 and pressed into sheets. Finally, spectra were recorded from 400 to 4000 cm^−1^, and 64 scans were averaged at a resolution of 4 cm^−1^ [44].

### 4.6. Pasting Property Evaluation

The potato powder pasting properties were analysed via a TecMaster Rapid Visco Analyser (RVA) (Perkin Elmer Instruments Co., Ltd., Shanghai, China), following previously described methods [45]. The potato powder (2.25 g) was suspended in 25 mL of water. Next, the suspension was equilibrated at 50 °C for 2 min, heated to 95 °C for 2.5 min at a heating rate of 12 °C/min, cooled to 50 °C at a cooling rate of 12 °C/min, and then held for 2 min. The stirring speed was set at 960 rpm in the initial 10 s and then reduced to 160 rpm. Finally, the pasting properties, including the peak viscosity, trough viscosity, breakdown, setback, final viscosity, and pasting temperature, were determined on the basis of the obtained viscographs.

### 4.7. Rheological Property Measurements

Briefly, 10 mL of water and 0.6 g of potato powder were added to a 50 mL centrifuge tube and heated at 95 °C for 20 min while stirring at 600 r/min. The samples were subsequently cooled to room temperature (25 °C) and placed on a parallel-plate system with a 40 mm diameter and a gap of 1 mm. Finally, an MCR301 rheometer (Anton Paar Inc., Shanghai, China) was used in the following assays [19,46,47].

#### 4.7.1. Strain Sweep Measurement

The strain sweep was evaluated at 0.01 to 10% strain on samples loaded onto the plate. The relative intensity and flow resistance of the potatoes were determined via the G′ and G′′ values.

#### 4.7.2. Frequency Sweep Measurement

The samples were loaded onto the plate. Next, angular frequency sweep measurements were conducted within the LVR at a strain amplitude of 1%, and between 0.1 and 100.0 rad/s. The variations in G′ and G″ were determined as a function of frequency.

#### 4.7.3. Steady Rheology Measurement

The flow curves were obtained by plotting the shear stress against shear rates of 0.1 to 100 s^−1^. Next, the flow behavior properties of the potato powder paste were estimated via the power–law model [46,47,48].

$$\sigma =Κ\cdot \gamma^{n-1}$$

where *σ* is the apparent viscosity (Pa∙s), *Κ* is the consistency coefficient (Pa·s ^n^), *γ* is the shear rate (s^−1^), and *n* is the flow behavior index.

### 4.8. Evaluation of the Texture Properties

The gel obtained through the RVA analysis was sealed with plastic wrap, cooled at 4 °C for 12 h, and left at room temperature for 1 h. Next, the TPA of the gel samples (potato gel cylinders, the height and diameter were 40 and 30 mm), including hardness, resilience, elasticity, adhesiveness, and chewiness, were determined via a Texture Analyser TA-XT2i (Stable Microsystem, Godalming, UK) with a P/36R probe. The testing conditions were as follows: distance, 10 mm; pretest speed, 1.5 mm/s; test speed, 1.5 mm/s; post-test speed, 1 mm/s; and trigger force, 5 g [49]. The experiments were conducted eight times.

### 4.9. Statistical Analysis

Unless otherwise stated, all experiments were performed independently in triplicate. The data are expressed as the means ± standard deviations. All the quantitative data were analysed via Duncan’s test and one-way analysis of variance (ANOVA) via IBM SPSS statistics 20.0 software at the 95% confidence level (*p* < 0.05). Pearson’s correlation coefficient and correlation diagrams were obtained via R software (version 3.2.1; Auckland, New Zealand).

## Figures and Tables

**Table 1:** Thermal and rheological properties (strain sweep and steady rheology) of FTD potato powder at different freezing temperatures. T_o_ is the onset melting temperature; T_p_ is the peak melting temperature; T_c_ is the conclusion melting temperature; ΔH is the melting enthalpy; LVR is the linear viscoelastic range; K is the consistency coefficient; n is the flow behavior index; and R^2^ is the coefficient of determination.

	T_o_ (°C)	T_p_ (°C)	T_c_ (°C)	ΔH (J/g)	LVR (%)	K (Pa·s ^n^)	n	R^2^
Control	65.21 ± 0.30 ^a^	68.75 ± 0.26 ^ab^	72.26 ± 0.27 ^c^	11.01 ± 0.18 ^b^	5.33 ± 0.30 ^a^	34.45 ± 0.31 ^a^	0.0823 ± 0.002 ^c^	0.9997
−5 °C	64.17 ± 0.11 ^cd^	68.21 ± 0.03 ^c^	72.24 ± 0.04 ^c^	12.17 ± 0.10 ^a^	2.23 ± 0.47 ^d^	8.96 ± 0.44 ^g^	−0.1015 ± 0.001 ^g^	0.9975
−10 °C	64.62 ± 0.37 ^bc^	68.83 ± 0.30 ^a^	72.88 ± 0.39 ^b^	11.87 ± 0.25 ^a^	3.06 ± 0.23 ^cd^	12.46 ± 0.40 ^f^	0.0656 ± 0.001 ^e^	0.9999
−15 °C	64.60 ± 0.29 ^bc^	68.74 ± 0.19 ^ab^	73.65 ± 0.20 ^a^	12.21 ± 0.28 ^a^	3.60 ± 0.28 ^bc^	13.49 ± 0.32 ^e^	0.0967 ± 0.001 ^b^	0.9986
−20 °C	64.25 ± 0.21 ^cd^	68.10 ± 0.30 ^cd^	72.34 ± 0.32 ^bc^	12.10 ± 0.59 ^a^	4.21 ± 0.33 ^b^	20.63 ± 0.17 ^b^	0.0428 ± 0.001 ^f^	0.9999
−35 °C	63.95 ± 0.24 ^d^	67.64 ± 0.35 ^d^	71.50 ± 0.34 ^d^	12.49 ± 0.45 ^a^	2.80 ± 0.43 ^cd^	17.41 ± 0.19 ^d^	0.0781 ± 0.001 ^d^	0.9990
−80 °C	64.78 ± 0.30 ^ab^	68.28 ± 0.34 ^bc^	71.89 ± 0.39 ^cd^	12.24 ± 0.27 ^a^	2.88 ± 0.67 ^cd^	18.45 ± 0.29 ^c^	0.1065 ± 0.002 ^a^	0.9997

Note: Different letters in the same column indicate significant differences (*p* < 0.05).

**Table 2:** Pasting properties of FTD potato powder at different freezing temperatures.

	Peak Viscosity (cP)	Trough Viscosity (cP)	Breakdown (cP)	Final Viscosity (cP)	Setback (cP)	Pasting Temperature (°C)
Control	3179.33 ± 37.71 ^ab^	1891.67 ± 27.81 ^b^	1287.67 ± 65.53 ^a^	2658.67 ± 174.89 ^c^	767.00 ± 147.08 ^c^	68.35 ± 3.54 ^a^
−5 °C	3556.67 ± 149.13 ^a^	3077.33 ± 97.22 ^a^	479.33 ± 19.82 ^bc^	4261.00 ± 53.16 ^b^	1183.67 ± 44.74 ^b^	70.83 ± 0.06 ^a^
−10 °C	3409.67 ± 106.45 ^ab^	3044.33 ± 125.33 ^a^	365.33 ± 20.29 ^bcd^	4682.67 ± 84.55 ^b^	1638.33 ± 53.56 ^a^	71.07 ± 0.38 ^a^
−15 °C	3352.67 ± 199.01 ^ab^	3002.67 ± 170.91 ^a^	350.00 ± 28.18 ^cd^	4646.00 ± 133.61 ^a^	1643.33 ± 75.09 ^a^	70.77 ± 0.06 ^a^
−20 °C	3131.33 ± 105.09 ^b^	2803.33 ± 99.90 ^a^	328.00 ± 5.89 ^d^	4397.67 ± 80.61 ^a^	1594.33 ± 28.19 ^a^	70.78 ± 0.02 ^a^
−35 °C	3389.00 ± 92.21 ^ab^	3071.67 ± 28.66 ^a^	317.33 ± 63.59 ^d^	4309.67 ± 34.57 ^b^	1238.00 ± 26.70 ^b^	70.77 ± 0.05 ^a^
−80 °C	3495.67 ± 295.07 ^ab^	3000.00 ± 185.81 ^a^	494.50 ± 113.91 ^b^	4257.00 ± 114.31 ^b^	1257.00 ± 71.50 ^b^	70.80 ± 0.04 ^a^

Note: Different letters in the same column indicate significant differences (*p* < 0.05).

**Table 3:** Textural properties of gels made from FTD potato powder at different freezing temperatures.

	Hardness (g)	Resilience (g)	Elasticity (g)	Adhesiveness (g)	Chewiness (g)
Control	53.00 ± 4.63 ^d^	20.03 ± 3.56 ^b^	92.09 ± 2.58 ^ab^	29.32 ± 2.81 ^b^	27.05 ± 3.28 ^b^
−5 °C	92.73 ± 5.70 ^bc^	19.44 ± 1.65 ^b^	91.07 ± 0.32 ^b^	53.30 ± 9.93 ^a^	48.52 ± 1.21 ^a^
−10 °C	100.30 ± 3.15 ^ab^	20.66 ± 5.09 ^b^	91.79 ± 1.11 ^ab^	53.27 ± 1.45 ^a^	49.37 ± 10.92 ^a^
−15 °C	107.34 ± 7.83 ^a^	23.85 ± 4.54 ^ab^	92.25 ± 2.99 ^ab^	55.96 ± 4.64 ^a^	52.25 ± 9.37 ^a^
−20 °C	103.72 ± 6.52 ^a^	24.84 ± 3.59 ^ab^	93.14 ± 1.64 ^ab^	56.85 ± 9.47 ^a^	52.16 ± 5.03 ^a^
−35 °C	93.85 ± 2.27 ^bc^	28.67 ± 2.09 ^a^	94.94 ± 2.37 ^a^	56.06 ± 2.31 ^a^	53.27 ± 3.51 ^a^
−80 °C	89.66 ± 5.84 ^c^	22.86 ± 3.24 ^ab^	91.05 ± 0.56 ^b^	49.46 ± 3.33 ^a^	45.03 ± 2.97 ^a^

Note: Different letters in the same column indicate significant differences (*p* < 0.05).

## Data Availability

The data presented in this study are available on request from the corresponding author.
